# A Novel Approach on Leukodepletion Filters: Investigation of Synergistic Anticancer Effect of Purified α-Defensins and Nisin

**DOI:** 10.34172/apb.2021.036

**Published:** 2020-04-19

**Authors:** Niloofar Sasani, Rasoul Roghanian, Giti Emtiazi, Afsaneh Aghaie

**Affiliations:** ^1^Department of Cell and Molecular Biology & Microbiology, Faculty of Biological Science and Technology, University of Isfahan, P. O Box 81746-79441, Isfahan, Iran.; ^2^Blood Transfusion Research Center, High Institute for Research and Education in Transfusion Medicine, Tehran, Iran.

**Keywords:** Defensin, Nisin, PC-3, HCT-116, Apoptosis

## Abstract

***Purpose:*** There are number of reports available regarding defensins activity against mammalian cells besides their antimicrobial and immune regulatory activities. This study aims to investigate anticancer and apoptosis activity of the purified defensins from leukodepletion filters alone or in synergism with bacterial peptide, nisin, on prostate and colorectal cancer.

***Methods:*** Leucoflex LCR-5 filters were backflushed by an optimized elution system. Isolated granulocytes were sonicated and the supernatant treated before further purification by high performance liquid chromatography (HPLC). SDS-PAGE and western blot testing verified the fraction. Cell culture on PC-3 (human prostate adenocarcinoma), and HCT-116 (human colorectal carcinoma) were conducted following by MTT assays in addition to annexin flow cytometry for sole and synergistic effects with peptide nisin.

***Results:*** Viable and active neutrophils could recover, and α-defensins were extracted and purified. Combinations of an optimal dose of α-defensins and nisin showed a remarkable synergistic effect on cancer cell lines (over 90% and 70% for PC-3 and HCT-116, respectively).

***Conclusion:*** It also observed that less than 40% of both cells could survive after co-treatment with optimal dose. Also, apoptosis was increased after treatment by these peptides together. Annexin Vpositive populations significantly increased in percentage in comparison with control.

## Introduction

Due to inappropriate use or misuse of therapeutics in medicine and veterinary medicine as the presence of many resistant genes in transferable plasmids to microorganisms, microbial resistance has been emerging over the past century. The fact has made it challenging to manage the microbial infection, and the effect of existing antibiotics has decreased. Thus, it seems necessary to innovate in the control of microbial infections. Of which is identification and development of antimicrobial peptides (AMPs). These are protected biomolecules throughout all living species that are involved in the fight against aggressive pathogens.^1^ An uncomplicated unspecific innate immunity system was rose billion years ago to defend microbial infections before evolving developed immune systems. It keeps being functional as the first defense barrier in all kingdoms of life.^2^ Such a system shall act as promptly cidal and be highly functional.^3^ The primary immune system is effective antimicrobially, in part, by low weight peptides with cationic properties that are active against Gram-positive and Gram-negative bacteria, fungi, parasites, and some viruses.^4^ Defensins and cathelicidin (LL-37) as two main groups of AMPs that store in azurophilic white blood cells and the secretion of these peptides by various epithelial cells in dermal and mucosal surfaces has been studied.^5^ AMPs also bested as capable molecules in immune processes such as inflammation and wound healing.^6^ Defensins widely spread within all the kingdoms of life. They manifest as multifunctional in human health and disease as well as performing several regulatory and antimicrobial missions. Based on their intramolecular disulfide bonds,^7,8^ they are categorized into three major types: α-, β-, and θ-defensins.^9-11^ As fusion to non-immunogenic tumor antigens, these peptides are prone to demonstrate or enhance antitumor immunity.^12,13^ These AMPs can also take place in signal transduction and regulation of the inflammatory effects, participate in wound healing and chemotaxis, control proliferation, and regulate the release of cytokines.^14^

Leukocytes depletion concept from blood was defined by Fleming, as early as 1920 when a simple system developed using a cotton wool plug in a bent glass tube with a constricted limb, associative to the structure of the modern filters.^15,16^ Leucocytes may cause severe reactions during transfusion. Furthermore, they can be selected by intracellular bacteria or pathogenic viruses as a confident asylum.^17-20^ Besides, leukoreduction filters have introduced a new source of natural human neutrophil peptide 1-3 (human AMPs available in human neutrophils’ granules) for research and development aspects.^21^ The leukoreduced filters contain quantities of healthy human blood cells.^22^ They demonstrate an economical source of neutrophils for α-defensins purification since they discard after use. Because of the strict regulation in screening for every single donation, the purified peptide is confident to be safe.

On the other hand, a growing number of studies have reported that α-defensins represent anticancer activity.^21^

Produced by *Lactococcus lactis*, nisin as a member of lantibiotics has widely recognized for its promising capability for clinical application as Generally Regarded as Safe(GRAS). This AMP was approved for use as a food preservative by the World Health Organisation (WHO) in 1969 and the US Food and Drug Administration (FDA) in 1988.^23^ Its primary use is related to its antibacterial activity. However, nisin has indicated selectivity towards cancer cells.^24^

Due to the toxicity of concurrent chemotherapeutics as well as attributed resistance, a need for novel anti-cancer remedies is sensed.^25,26^ This study aims to evaluate anti-cancer activity and apoptosis potentiality of human alpha-defensins, extracted and purified from polymorphonuclears (PMNs), which were isolated from recovered leukocytes trapped in Leucoflex LCR5 filters. The selected cell lines are PC-3 (human prostate cancer cell line), and HCT-116 (human colorectal cancer cell line), and the study is also to be conducted for assessment of any possible synergistic activity of this purified defensins, together with nisin in both cell lines.

## Materials and Methods

### Neutrophil recovery from leukoreduction filters

Leucoflex LCR-5 filters (Macopharma, France) are consumed by the Iranian Blood Transfusion Organization (IBTO) to process leukodepleted products. Recovery of the cells was conducted for eight filters using an optimized method that was developed by the team. The system was previously validated to recover the optimum yield of viable granulocytes from Leucoflex LCR-5 filters. The core concept was to construct a mechanical system to decline human manipulation on filter elution. It consisted of an adjustable three-way connector, a peristaltic pump, and an air blower. Optimized elution buffer of PBS (pH=7.2) containing 2 mM EDTA and 4% (w/w) dextran 40 was pressed into the filters from the opposite way of routine with a determined volume and pressure.

In brief, 150 milliliters of the obtained suspension applied in leukocyte recovery. Gradient centrifugation with Histopaque^®^ (Sigma-Aldrich, USA) used to eliminate mononuclear cells. the suspension was added gently to the Histopaque^®^ (5:2) using a pipette Pasteur. Then it was centrifuged at 800 g for 20 minutes. The upper layers were aspirated, and the last segment of PMNs was diluted 1:1 by Hank’s balanced salt solution (HBSS^®^, Gibco Co, USA). Red blood cell deletion was carried out by high-fraction dextran sedimentation following a step of hypotonic lysis. In 50-mL sterile falcons, the suspension was mixed with 6% dextran containing 0.9% NaCl (1:1) and remained at room temperature for one hour. The upper transparent layer (which consisted of neutrophils), was collected. Then cold deionized water was added to the collection for 30 seconds followed by a 30 second period of PBS addition to the medium. This procedure continuously repeated until all the red blood cells cleared. Red blood cells cannot tolerate hypotonic situations, while PMNs can survive. Extracted PMNs then assessed for their viability using trypan blue 0.4% under an optical microscope.

A 24 wells plate considered to encounter PMNs with platelets in the HBSS medium. A well filled with sole recovered PMNs as the negative control. An average number of 10^6^ cells per milliliter was adjusted, and each well filled with 200 μL of the sample diluted by HBSS. 200 μL of platelets (10^8^ cells/mL) was added to each well together with 200 μL of RPMI medium (Gibco, USA). After homogenization, the plate inserted into a CO_2_ incubator for 60 minutes. Each well was added by 200 μL of phosphate buffer to assess the neutrophil-platelet complex formation and was pipetted for multiple times for entire well containment to disserve from the bottom. Each well was then transferred to a sterile microtube and centrifugated in 970 g for 15 minutes in a refrigerated device. Supernatant was aspirated, and the pellet was studied by Giemsa staining to evaluate phagocytosis activity of the recovered neutrophils.

Having isolated PMNs from the filter eluted suspension and washed by PBS, 10^8^ cells mixed with a milliliter of 0.34 M sucrose solution (pH=7.4). Cells lysed in the presence of ice, three times and each time for 30 seconds in the assistance of sonication. When sonication finished, elution centrifugated in 200 g for 15 minutes at 4°C, and cell extract was collected. It faced another 30 minutes of centrifugation in 500 g at 4°C. Supernatant, consisted of intragranular substances was solved in 0.34 M sucrose solution, lyophilized, and used for further steps.

### Sodium dodecyl sulphate-poly acrylamide gel electrophoresis (SDS-PAGE) analysis

A small amount of lyophilized sample was dissolved in sterile distilled water. The sample and commercial HNP-1 (Abcam, USA) were loaded on a 15% SDS gel and analyzed at 110 V for 2:30 hours. The system ran, and the results assessed after illustration.

### Peptide purification

A portion of the lyophilized sample was dissolved in sterile distilled water, and treated by steps of 0.25 and 0.45-micrometer filtration systems (Sartorius, Germany), and then 3 KD falcon filters (Sartorious, Germany) used to achieve a size-specific peptide elute as the sample for further purification. The main purification step conducted by a semi-preparative high performance liquid chromatography (HPLC) system (Shimadzu, Japan). Due to pretreatments and not to manipulate peptide charge, a 60 cm size exclusion column (Tosoh Biosciences, Japan) chosen for cycle run. The sample injected into the system in 2 mL volumes and a buffer method was defined based on 34% acetonitrile, and 0.1% THF without any gradient. The system was set to elute at 0.75 mL/min, and picks at 280 nm were fractionated in the vials. A sample of the commercial product ran by the same system, and the reference graph has already prepared.

### Western blot analysis

The presence of the peptide verified through subsequent western blotting. Using a semi-dry system (Bio-Rad, England), dissolute lyophilized fractions from HPLC transferred to a polyvinylidene difluoride membrane, and it was incubated overnight with 5% skim milk in PBS as blocking buffer. After washing, the membrane remained in an incubator while added with HNP 1-3 monoclonal antibody dilution (1:100) (Hycult Biotechnology, England) for 2 hours. Passing an extra PBS washing step, 1:3000 diluted goat anti-mouse IgG horseradish peroxidase-conjugated antibody added. Then the membrane experienced one hour more incubation time at room temperature. Protein bond indicated by ChemiDoc (Bio-Rad, Hercules, USA).

### Cell culture

PC-3 cell line purchased from the Iranian Biological Resource Center (IRBC, Tehran, Iran). The cell lines were cultured in supplemented RPMI- 1640 media (10% FBS, 2 mM glutamine, 100 U/mL penicillin, and 100 U/mL streptomycin), and incubated at 37°C under 5% CO_2_. On the other hand,HCT-116 cell line (IRBC, Tehran, Iran) was cultured in enriched RPMI-1640 media with 10% FBS and 2 mM glutamine, and incubated at 37°C and 5% CO_2_ as well. Both cell lines exposed to 5, 10 and 20 μg/mL of purified alpha defensin. Also, cell lines were treated with nisin 25, 50, 75 μg/mL.

### MTT assay

In vitro cell viability was determined byMTT assay. For this, a 96-well plate used for cell line culture cells. After a 24-hours incubation time, MTT assays conducted. The cells added by 0.025 mM phenazine methosulphate, and MTT solution (Sigma, Germany) and following incubation for three hours, the optical density (OD) at 570 nm was measured.

### Synergistic cytotoxicity assay

The cytotoxicity of purified defensin and nisin on mammalian cells examined using human prostate and colon cancer, which were cultured with RPMI medium (Gibco, UK) supplemented with 10% FBS, 10 mM l-glutamine, 10 ng/mL EGF and 1% penicillin-streptomycin; and 5 ×10^3^ cells/well. After 24-hour incubation at 37^◦^C with 5% CO_2_ within 12-hour starvation performed. The best doses of the two peptides and half of their best doses concentrations (7 µg/mL for HNP 1-3 and 35 µg/mL for nisin) added and, cells were treated for 24 hours, before MTT incubation for four hours. The growth medium was separated, and 100 μL of DMSO added for the formazan crystals to dissolve. The OD then read at 570 nm. 1% Triton X-100 removed all control treatments.

### Annexin V/propidium iodide (PI) staining

1× 10^6^ cells were plated into each well of 6-well plates and mixed up with different concentrations of α-defensin (5, 10, and 15 µM) and nisin (25,50 and 75 µM) or any peptides addition. After being incubated for 24 hours, cells were harvested and eluted twice with cold PBS and reconstituted in 1× binding buffer. Cells were then added to a tube of 1.5-ml previously contained with 100 µL of binding buffer and 5 µL of FITC-conjugated Annexin V. Afterwards, PI included. Following a gentle vortex, cells then incubated for 15 min at room temperature in the dark. Stained cells were counted by flow cytometry (Sysmex Partec, Germany) after the addition of 400 µL of 1× binding buffer, and FloMax software used for the analysis of the results.

## Results

### α-Defensins are efficiently isolated and purified from numerous recovered viable neutrophils

**Figure 1:**
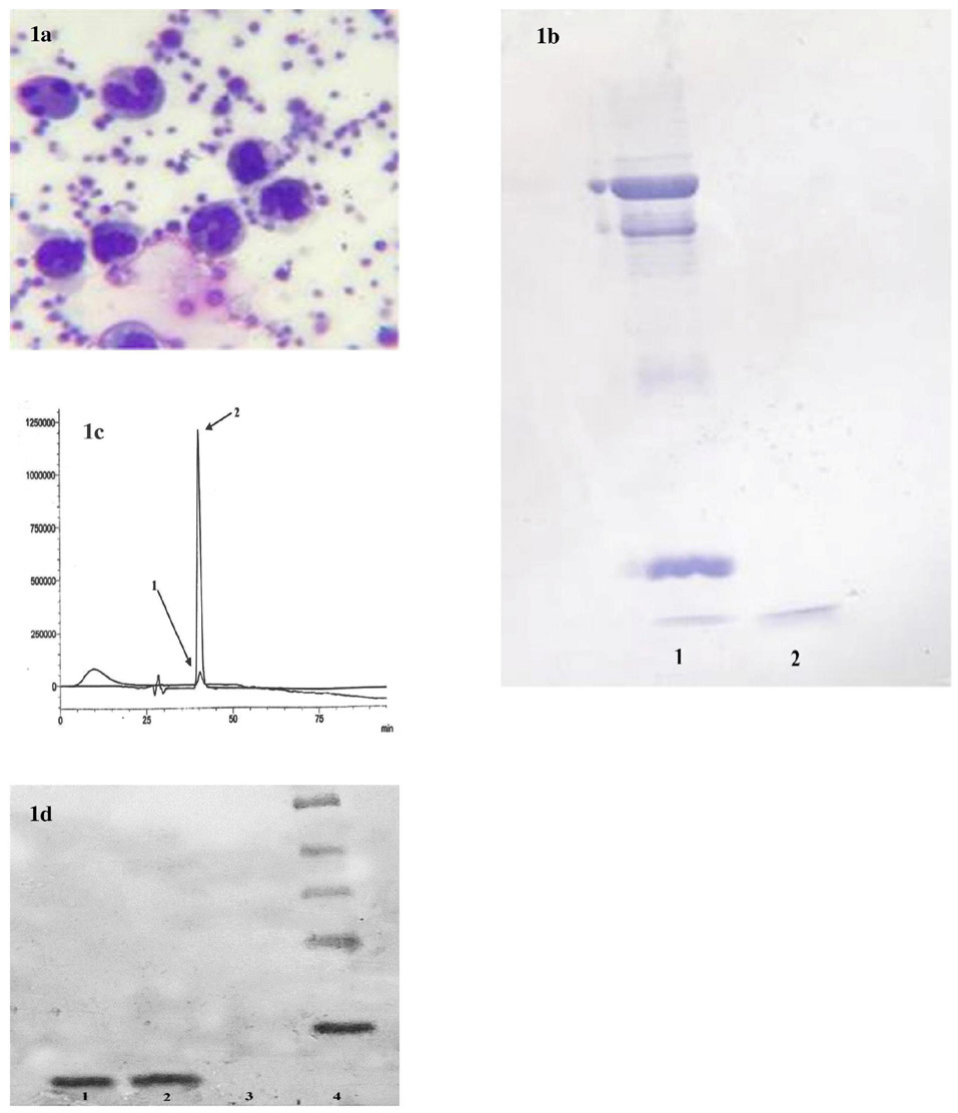
**(a)** Activity of polymorphonuclears isolated from leukoreduction filters. Recovered cells could be active in phagocytosis of platelets. (**b)** SDS-PAGE analysis. 1) sample of commercial HNP-1 (Abcam, USA) and 2) sample of granule lysate from isolated neutrophils. The test confirmed presence of defensins in cell lysate obtained from the neutrophil granules isolated from leukoreduction filters. 9**c)** HPLC Chromatogram. 1) commercial alpha defensin. 2) Purified HNP from recovered neutrophils. High amounts of HNP 1-3 could be purified from eight leukoreduction filters. (**d)** Western blot analysis of purified HNP 1-3. The presence of α-defensins at the expected masses (3.4 kDa) was detected in the eluates (2). The fraction obtained from HPLC column that were unbound proteins (not HNP 1-3) used as the negative control (3). (1) was bound commercial α-defensin 1 (Abcam, USA).

Human neutrophils display antimicrobial activity, which is understood to originate from its granules. Among granular components and mostly in azurophilic granules, human neutrophil α-defensins (HNP, isoforms 1–4, which differ in a single N-terminal residue but have similar antimicrobial properties) and cathelicidin have been well-described. HNP-1 chose as a proper marker for the study due to its constitution of 30% in azurophilic granules.^27^ An average number of 6.27 × 10^8^ neutrophils were recovered and isolated from each Leucoflex LCR-5 filter by our self-developed mechanical system. Over 97% of them showed viability, and they were thoroughly able to phagocyte platelets (Figure 1a), which confirmed functional activity of the recovered neutrophils.

In contrast with commercial HNP 1-3 bond on SDS gel, the sample obtained from granular lysate demonstrated a sharp, bold bond (Figure 1b). The bond represented a high concentration of α-defensinsin the prepared sample. The granule lysate was passed through steps of filtration and gel permeation column to separate the components. The second fraction showed exact chromatographic position to the standard at approximately 40 minutes of the mobile phase running with a sharp pick of 1.26 V, which was about 250 times sharper than the commercial sample pick (Figure 1c). Commercial sample prepared in 200 ppm. The fraction was analyzed by western blotting for further verification (Figure 1d).

### α-Defensin and nisin have a synergistic anti-tumor effect on prostate and colorectal cancer cell lines

**Figure 2:**
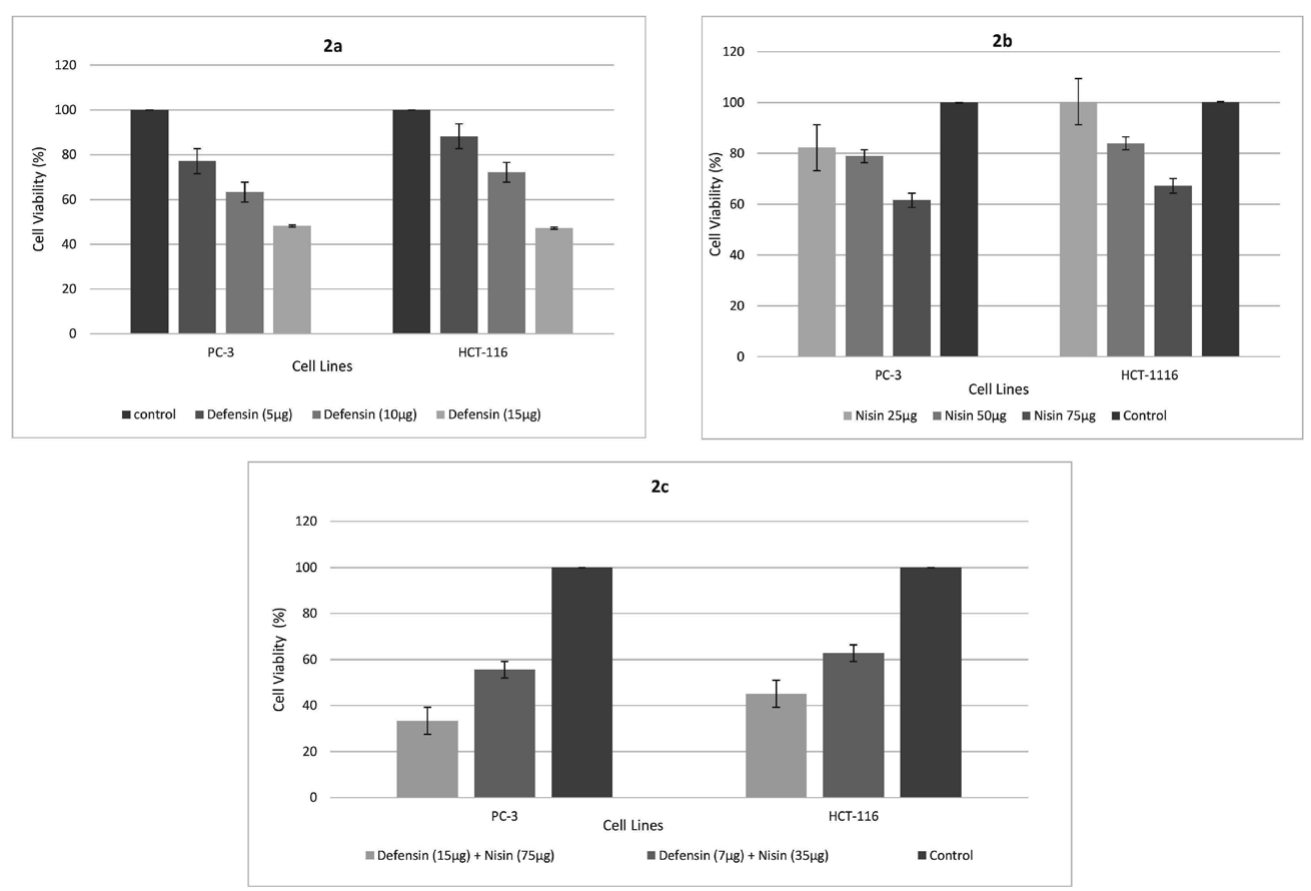
The effect of purified α-defensin on HCT-116 and PC-3 cell viability (**a**). Cells were treated with different concentrations (5, 10 and 15 µg/mL) of α-defensin for 24 hours and cell viability was measured by the MTT assay. The statistical analysis was performed using Student’s t-test for samples and differences were considered significant when *P* < 0.05. The effect of nisin on HCT-116 and PC-3 cell viability (**b**). Cells were treated with different concentrations (25, 50 and 75 µg/mL) of nisin for 24 hours and cell viability was measured by the MTT assay. The statistical analysis was performed using Student’s t-test for samples and differences were considered significant when *P* < 0.05. Co-treatment with purified α-defensin and nisin shows a remarkable synergistic effect on cancer cell lines. The effect of α-defensin – nisin (15-75 µg/mL and 7-35 µg/mL) on HCT-116 and PC-3 (**c**) cell viability is represented in the graph. Cells were treated for 24 hours and cell viability was measured by the MTT assay. The statistical analysis was performed using Student’s t-test for samples and differences were considered significant when *P* < 0.05.

Cell viability reached approximately 60% and 70% in PC-3 and HCT-116, while purified α-defensin was at the concentration of 10 µg/mL. Yet, the peptide could decrease both cell lines’ viability by 50% at the strength of 15 µg/mL (Figure 2a). It demonstrated that nisin could only influence by 40% on viability at its best dose of 75 µg/mL. However, this effect displayed more on PC-3 than HCT-116 cell line (Figure 2b). A combination of purified α-defensin and nisin showed a remarkable synergistic effect on cancer cell lines. Co-treatment with 15 µg/mL defensin and 75 µg/mL nisin showed over 60% lethal effect on PC-3 and approximately the same on HCT-116 cells. It also observed that 60% of both cell lines could survive after co-treatment with 7 µg/mL purified HNP 1-3 and 35 µg/mL nisin (approximately half of the best effective doses of the biomolecules) (Figure 2d). The experiment conducted three times and, information introduced as a mean ± standard deviation. The student’s t-test was run for statistical analysis and interpreted as significant difference when *P* < 0.05.

### α-Defensin and nisin induce apoptosis in prostate and colorectal cancer cell lines

**Figure 3:**
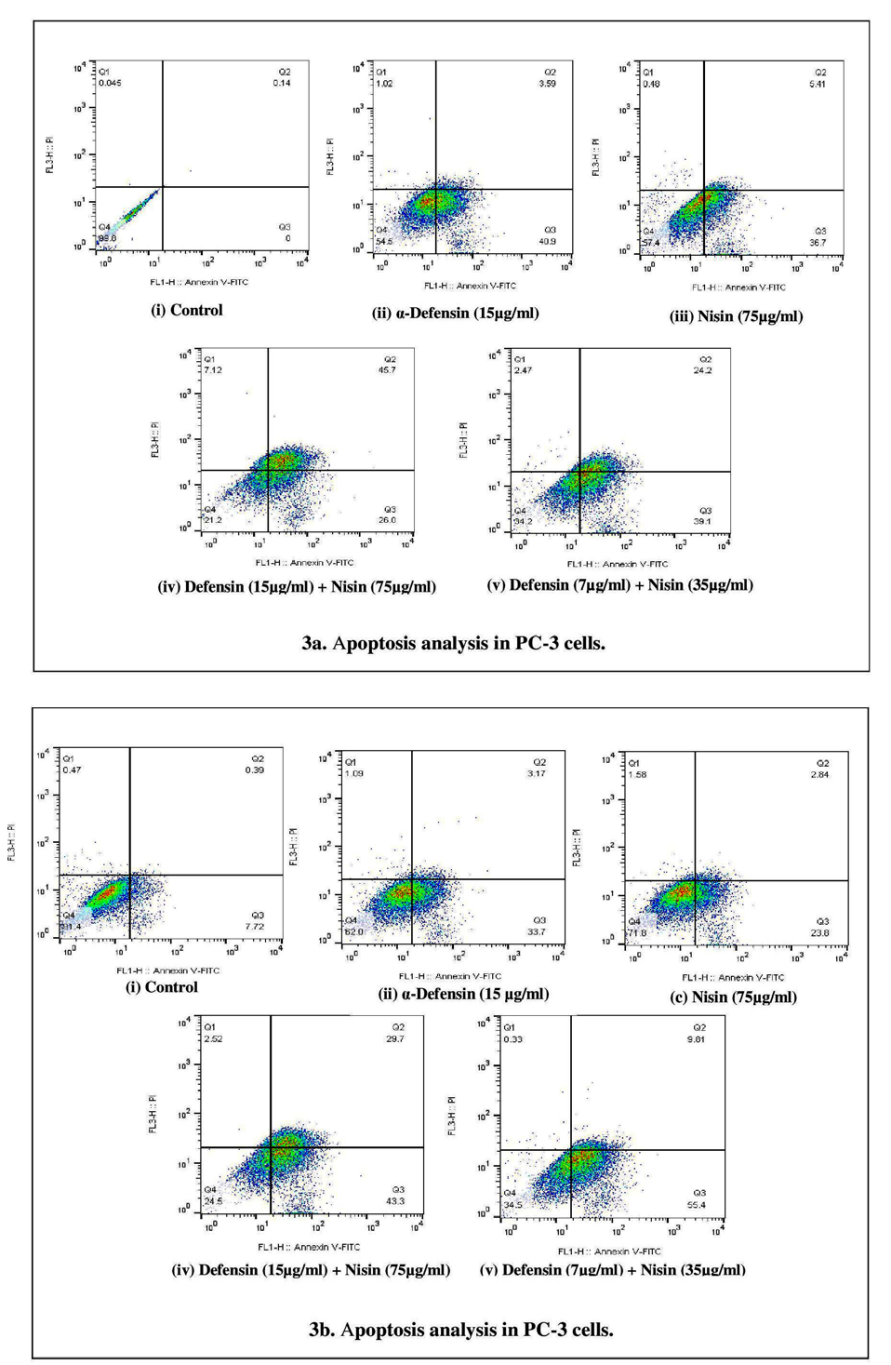
**A**poptosis analysis in PC-3 (**a**). Flow cytometric analyses of the prostate cancer cells treated with best concentration of control (i), purified α-defensin (ii), nisin (iii), best effective dose in co-treatment (iv) and best half effective dose concentration (v). The cells were stained with annexin V-FITC and PI and subjected to flow cytometry. The distribution of the cells is presented as the percentage of total cells counted. **A**poptosis analysis in HCT-116 (**b**). Flow cytometric analyses of colon cancer cells treated with best concentration of control (i), purified α-defensin (ii), nisin (iii), best effective dose in co-treatment (iv) and best half effective dose concentration (v). The cells were stained with annexin V-FITC and PI and subjected to flow cytometry. The distribution of the cells is presented as the percentage of total cells counted. The statistical analysis was performed using one-way ANOVA test for samples and differences were considered significant when *P* < 0.05.

Apoptosis was investigated by Annexin V/PI staining, while cancer cells were treated by sole purified α-defensin (15 µg/mL), sole nisin (75 µg/mL), and combinations of these two peptides at their best an half best effective doses (15-75 µg/mL and 7-35 µg/mL). The population of annexin V positive (apoptotic) PC-3 cells declined significantly (about 40%) comparing to the control, while treated by best doses of purified HNP 1-3 or nisin. The crowd experienced a different count in HCT-116 cells with 25% and 35% at the presence of α-defensin and nisin, respectively. Annexin V/PI positive (late apoptotic) cells touched maximum in PC-3 cells with about 45% at the best dose co-treatment of the peptides, the same amount for Annexin V positive/PI negative population in HCT-116 cells in the same condition. This cell line faced a growing percentage of 50% in the early apoptotic population in the presence of 7 µg/mL purified α-defensin/35 µg/mL nisin while the population of the late apoptotic cells reached to 10%. At the same concentration of co-treatment, the early and late apoptotic cells population was about 60% in PC-3 cells. In general, the co-treatment of 15 µg/mL of our purified α-defensin and 75 µg/mL of peptide nisin significantly induced apoptosis (approximately 70%) in PC-3 and HCT-116 cell lines, compared to the control (Figure 3a and 3b).

## Discussion

In this study, we could set a shortcut for purification of the human neutrophil peptide, defensin, from neutrophils recovered from leukoreduction filter Leucoflex LCR5. Also, we investigated the anticancer activities of this peptide on PC-3 and HCT-116 cell lines as well as its synergistic anticancer activity with natural poly-peptide, nisin, which was our novelty of work. Although many studies have evaluated the association between carcinogenesis and AMPs,^28^ the results have been inconsistent. α-defensins could promote cancer growth^29^ whereas there are other reports to demonstrate α-defensins cytotoxicity.^30-32^ The outcome of our study severely defends the particular cytotoxicity effect of α-defensins. Despite many reports on cytotoxic effects of HNP-1,^33,34^ our study indicated a markedly potent anticancer activity for α-defensins together with nisin comparing α-defensins alone. It is an initiation of the influence of α-defensins and nisin on mammalian cell lines. Defensin induced cell death at its high concentrations (15 mg/mL). Nisin addition to α-defensins in the cell culture resulted in a remarkable decline in the effective concentrations. Here represented that nisin and α-defensins showed a synergistic effect against those two cancer cell lines. This phenomenon makes this combination be of concern in forming new bullets to defend colorectal and prostate cancers. Public health is continuously burden by cancer for estimated 8.7 million deaths per annum. Tremendous progress has achieved in declining cancer mortality rates, and upgraded movements have determined in therapeutic protocols within the past few years. However, novel approaches to therapeutic development are still an urgent priority in the advanced setting of treatment-refractory malignancies. Although there are overwhelming clinical successes of cancer immunotherapy such as immune checkpoint inhibitors (e.g., pembrolizumab, ipilimumab, atezolizumab) against multiple tumor histologies, it is inevitable whether the development of novel immune-modulatory strategies is of deep concern in oncology treatment.^35,36^

Former reports have demonstrated strong concerns over natural defensins purification and study. Novel peptide-based drugs might be alternatives. Previous reports pointed out that FF/CAP18 (an analog for peptide cathelicidin) is susceptible to apoptotic cell death on SAS-H1,^37^ and HCT-116 cell lines.^38^ Addressing the fact, a novel approach for defensins extraction and purification has been developed by the team based on biologic recovering of leukoreduction filters used in transfusion medicine. The results of this study found that after α-defensin and nisin treatment, apoptosis was significantly expanded compared *in vitro*.

## Conclusion

With the highest incidence rate in men, prostate carcinoma places as the third leading criterion in cancer mortality after lung and colorectal cancers.^39^ Colorectal cancer ranks three among the most diagnosed malignancies in males and two in females.^39,40^ Currently, hormone therapy, surgery, or irradiation are options for prostate cancer treatment. The availability of therapeutic arsenal could not yet defend against cancer cells since the response is not well enough to single or multiple drug regimens. Also, adverse effects are significantly harmful. There are limited numbers of AMPs extracted up to the date. Apoptotic synergism is confirmed by this study, however, more details in mechanism of apoptosis in prostate and colorectal cancers shall be revealed to reach novel therapeutic pathways. On the other hand, further *in vivo* studies as well as oral peptide delivery systems could also be of a crucial significance in cancer therapy.

## Ethical Issues

All applicable international, national, and, or institutional guidelines for the care and use of animals were followed.

All procedures performed in studies involving human participants as per the ethical standards of the institutional and national research committee (IR NIMAD REC 1396 294) and with the 1964 Helsinki declaration and its later amendments.

## Conflict of Interest

The authors declare that they have no conflict of interest in this study.

## Acknowledgments

The work funded by the National Institute for Medical Research Development Grant No. 962524. Authors would acknowledge the University of Isfahan (Isfahan, Iran) and High Institute for Research Education in Transfusion Medicine (Tehran, Iran) for their extra financial supports. Authors would also like to appreciate Dr. Seyed Mehrdad Jalali (Iranian Blood Research and Fractionation Co., Tehran, Iran) great assistance in peptide purification and cell culture method design for this study.
